# Infection prevention and control in ultrasound - best practice recommendations from the European Society of Radiology Ultrasound Working Group

**DOI:** 10.1007/s13244-017-0580-3

**Published:** 2017-11-27

**Authors:** Christiane M. Nyhsen, Hilary Humphreys, Roland J. Koerner, Nicolas Grenier, Adrian Brady, Paul Sidhu, Carlos Nicolau, Gerhard Mostbeck, Mirko D’Onofrio, Afshin Gangi, Michel Claudon

**Affiliations:** 1 Radiology Department, City Hospitals Sunderland, Kayll Road, Sunderland, SR4 7TP UK; 2 Department of Clinical Microbiology, The Royal College of Surgeons in Ireland, Dublin, Ireland; 3 Department of Microbiology, Beaumont Hospital, Dublin, Ireland; 4 Infection Prevention and Control Department, Department of Microbiology, City Hospitals Sunderland, Kayll Road, Sunderland, SR4 7TP UK; 5 Service d’Imagerie Diagnostique et Interventionnelle de l’Adulte Groupe Hospitalier, Pellegrin Place Amelie Raba-Leon, 33076 Bordeaux, Cedex France; 6 Department of Radiology, Mercy University Hospital, Grenville Place, Cork, T12 WE28 Ireland; 7 King’s College Hospital, Denmark Hill, London, SE5 9RS UK; 8 Clinic Villarroel 170, 8036 Barcelona, Spain; 9 Wilhelminenspital, Montleartstr. 37, 1160 Vienna, Austria; 10 Radiology, Policlinico G.B. Rossi, VERONA, piazzale LA SCURO 10, 37134 Verona, Italy; 11 NHC, 1, Place de l’Hôpital, 67091 Strasbourg, France; 12 Children Hospital, University Hospital - Nancy Brabois, Rue du Morvan, 54511 Vandoeuvre Les Nancy, Cedex France

**Keywords:** Ultrasound, Infection prevention and control, Disinfection, Patient safety, Guidelines

## Abstract

**Objectives:**

The objective of these recommendations is to highlight the importance of infection prevention and control in ultrasound (US), including diagnostic and interventional settings.

**Methods:**

Review of available publications and discussion within a multidisciplinary group consistent of radiologists and microbiologists, in consultation with European patient and industry representatives.

**Recommendations:**

Good basic hygiene standards are essential. All US equipment must be approved prior to first use, including hand held devices. Any equipment in direct patient contact must be cleaned and disinfected prior to first use and after every examination. Regular deep cleaning of the entire US machine and environment should be undertaken. Faulty transducers should not be used. As outlined in presented flowcharts, low level disinfection is sufficient for standard US on intact skin. For all other minor and major interventional procedures as well as all endo-cavity US, high level disinfection is mandatory. Dedicated transducer covers must be used when transducers are in contact with mucous membranes or body fluids and sterile gel should be used inside and outside covers.

**Conclusions:**

Good standards of basic hygiene and thorough decontamination of all US equipment as well as appropriate use of US gel and transducer covers are essential to keep patients safe.

***Main messages*:**

*• Transducers must be cleaned/disinfected before first use and after every examination.*

*• Low level disinfection is sufficient for standard US on intact skin.*

*• High level disinfection is mandatory for endo-cavity US and all interventions.*

*• Dedicated transducer covers must be used for endo-cavity US and all interventions.*

*• Sterile gel should be used for all endo-cavity US and all interventions.*

## Introduction

A recent publication has shown that bacterial contamination of ultrasound (US) transducers is significantly higher than contamination of public toilet seats and bus poles [1]. Once any surface is contaminated, pathogens can survive a prolonged period of time [2, 3]. Even in proven cases of infection transmission, the exact route may remain unclear and insufficiently decontaminated needle guides as well as post-procedures follow-up US examinations without high level decontamination merit consideration [4, 5]. This highlights the need for thorough standardised decontamination protocols.

National guidance and legislation regulating decontamination procedures vary throughout Europe. European guidance on interventional US procedures has already been published stressing amongst other issues the importance of good hygiene [6]. However, detailed European guidance on transducer decontamination, choice of US gel and transducer covers is lacking. There remains a wide range of practice amongst European US practitioners, as shown in a survey of the European Society of Radiology (ESR) US Working Group (WG) [7].

## Methods

Radiologists from the ESR US WG, together with expert microbiologists, formed a multi-disciplinary group who as a first step undertook the survey mentioned above [7]. This identified considerable variations in practice and apparent confusion as to what is best practice.

A detailed literature review of available US-specific evidence was carried out with a variety of PubMed searches of publications from 1990 to 2017, including international and national surveys and guidelines, observational studies, case reports and opinion pieces. In the absence of research systematically addressing the specifities of US procedures and their respective environments and the presence of such a heterogeneous evidence base, it was not possible to grade the evidence or to indicate the strength of existing guidelines. Therefore, the decision was taken to formulate the best practice recommendations hereby presented.

These recommendations have been derived from reviewing the evidence base as obtained above and applying key principles of prevention of cross-infection in the healthcare setting where there is no published specific evidence derived from the US environment. Subsequently, these consensus recommendations were discussed and agreed by the WG members who undertook this task, stressing that they need to be incorporated into local guidelines and must be compliant with respective national legislation. Transducers can be vectors of infection transmission with most serious outbreaks relating to endoscopic US procedures [8–11]. Risk evaluations have been attempted but subsequently disputed; hence, the exact risks will remain uncertain [12–14].

Evidence shows that adequate protocols combined with staff training can achieve efficient disinfection [1]. It is the hope of the ESR US WG that publication of these recommendations, despite their limitations in terms of the evidence base, will raise awareness and improve the training of all US practitioners, thus ultimately contributing to improvements in patient safety.

## Transmission of infection through US procedures

In principle, once any surface has been colonised, pathogens can survive for periods of time longer than many might expect [2]. This applies in particular to synthetic materials including US transducer surfaces and other parts of the US equipment. Survival times on dry inert surfaces of bacteria such as *Escherichia coli*, *Pseudomonas aeruginosa *and *Staphylococcus aureus*, including methicillin-resistant *S. aureus* (MRSA) can be several months or longer, and that of viruses such as hepatitis A, herpes simplex virus (HSV) and rotaviruses several weeks (Table 1). Even fungi such as *Candida albicans* can survive up to 120 days. In addition, post-contamination survival will be even longer with co-existent organic material such as skin cells or body fluids, providing a protective nidus for microbes which even disinfectants may not fully penetrate.

**Table 1:** Survival of pathogens on dry inanimate surfaces (shortened from Kramer et al. BMC Infectious Diseases 2006)

Type of pathogen	Duration of persistence
Bacteria:	
*Campylobacter jejuni*	up to 6 days
*Clostridium difficile* (spores)	5 months
*Escherichia coli*	1.5 h – 16 months
*Haemophilus influenzae*	12 days
*Mycobacterium tuberculosis*	1 day – 4 months
*Neisseria gonorrhoeae*	1–3 days
*Pseudomonas aeruginosa*	6 h – 16 months (dry floor up to 5 w)
* Staphylococcus aureus*, including MRSA	7 days – 7 months
Fungi:	
*Candida albicans*	1–120 days
Viruses:	
SARS associated virus	72–96 h
HAV	2 h – 60 days
HBV	> 1 week
HIV	> 7 days
Herpes simplex virus 1 & 2	4.5 h – 8 weeks
Papillomavirus	16 > 7 days
Rotavirus	6–60 days

Ultrasound examinations and procedures carry different risks depending on the likelihood of exposure to the normal bacterial flora of patients (as discussed below), contact with body fluids and the degree of invasiveness of the US procedure. Adopting a modified Spaulding classification^1^ [15], procedures can be classified into:

Non-critical: non-invasive, contact of US transducer with intact skin only, requiring low level disinfection.

or

Critical: invasive, such as US-guided punctures or injections, contact of the US transducer with mucous membranes and body fluids, or a combination of both.

The reasoning for these recommendations is as follows:

US-assisted invasive procedures range from minimal invasive fine needle aspirations to endoscopic and intra-operative use of US. When assessing the risk of transmission of infective agents, all these procedures have in common the breaching of the intact skin or mucous membranes. Taking acupuncture as an example of a minimally invasive procedure: fine acupuncture needles have been demonstrated to carry viral material after treatment of hepatitis C-positive patients [16]. In cases of directly US-assisted punctures, contact of the transducer with infected materials cannot be excluded. Consequently any US-assisted invasive procedure or any procedure potentially causing micro-trauma to the skin or mucous membranes has to be categorised as critical.

The category “semi-critical” as detailed in the Spaulding classification describes devices that are in contact with intact mucous membranes of non-sterile body sites such as the vagina. Because the integrity of these mucous membranes cannot be taken for granted and procedure-associated micro-trauma can never be excluded, this category has been omitted. The generally accepted recommendations for disinfection are similar to those for critical procedures, i.e., transducers require high level disinfection (HLD) or sterilisation.

## Potential microbes causing US-related invasive infection

### Normal human microbial flora

The skin and almost every epithelial layer of the human body are colonised with a physiological bacterial flora, which varies from site to site. Healthy individuals may also carry potential pathogens, e.g., up to 20% of the healthy population carry* Streptococcus pyogenes* (Group A streptococcus) and/or *Staphylococcus aureus* in their throat. Healthy individuals may carry toxigenic strains of *Clostridium difficile* or even *Salmonella typhi*, the most famous example being Typhoid Mary [17]. With the exception of herpes viruses, human papilloma virus (HPV) and some others, humans do not carry viruses.

A distinction should also be made between “endogenous infections”, which may occur when microbes of the patient’s normal flora enter normally sterile spaces, from those referred to as “exogenous infections”, when pathogens are introduced from outside the patient, i.e., from other patients, healthcare workers or the inanimate environment. The risk of endogenous infections for example is unavoidable in the case of trans-rectal ultrasound-guided biopsies, where the needle may introduce microbes from the normal rectal flora into the normally sterile prostate/peri-prostatic space [18, 19]. This would be different from a previously known hepatitis C virus (HCV) negative patient who presents with acute viral hepatitis following an ultrasound-guided procedure, where this new infection is caused by a pathogen most likely acquired from another patient [4, 5].

Individuals who are asymptomatic carriers of potential pathogens may have developed a degree of immunity and be less susceptible to develop an infection caused by their endogenous flora. However, if these organisms are transmitted to another patient, through a contaminated US transducer or by other means, they may cause an infection, and are therefore classified as “potential pathogens”. An example is *S. aureus*, which is carried by up to 30% of healthy individuals in the nose, and which may cause post-surgical wound site infections.

The physiological flora and potential pathogens vary from site to site (Table 2).

**Table 2:** Body sites and their physiological flora and potential pathogens

	Normal flora	Potential pathogens	Pathogens
Skin	Coagulase-negative Staphylococcus spp. (*S. epidermidis* etc.), diphtheroids, Gram-positive and Gram-negative anaerobes	*S. aureus*	
Throat and upper airways	*Viridians streptococci*, Neisseria spp., Gram-positive and Gram-negative anaerobes	*S. aureus, S. pyogenes, Haemophilus. influenzae, N. menigitidis*	*Mycobacterium tuberculosis*
Gastrointestinal tract	*Escherichia coli* and related Gram-negative bacilli,* Pseudomonas aeruginosa, Enterococcus faecalis* and other Enterococcus spp., *Clostridium perfringens*, *C. difficile*, and other Clostridium spp., Gram-positive and Gram-negative anaerobes	*Salmonella typhi* and other Salmonella spp., Campylobacter spp., Shigella spp., pathogenic strains of E.coli such as *E.coli O157, C.difficile*	
Male perineum and external genitalia	Faecal and skin flora as outlined above, Candia spp.	*S. aureus*, *E. coli*	Sexually transmitted pathogens such as *N. gonorrhoeae, Chlamydia trachomatis* and other Chlamydia spp., *Mycoplasma genitalis,* HSV I + II
Female perineum, external genitalia and vagina	Faecal and skin flora as outlined above, Candida spp., Lactobacillus spp.	*S. aureus, E. coli*	Sexually transmitted pathogens such as *N. gonorrhoeae, Treponema pallidum, Chlamydia trachomatis* and other Chlamydia spp., *Mycoplasma genitalis*, HPV, HSV I + II
Body fluids including blood			Blood borne viruses, i.e. hepatitis B virus, HCV, human immunodeficiency virus

Please note: These are examples. It is not an exhaustive list

## Definition of decontamination procedures

### Cleaning

Removal of dirt and any visible materials thus rendering items macroscopically clean. The use of detergents will remove most viable bacteria. Thorough cleaning must always precede any disinfection or sterilisation procedure. Otherwise, they are likely to be ineffective as the presence of protein or other material prevents the penetration of the disinfectant or sterilant to the surface to be cleaned.

### Disinfection

Inactivation of most viable bacteria. This will include most pathogens likely to be transmitted via US procedures. Resistance to antibiotics does not correlate with resistance to disinfectants or biocides. However, there is some concern that the overuse or abuse of disinfectants may lead to resistance. Pathogen survival is dependent on inoculation size and the presence of protein. The latter protects microbes from the action of disinfectants; hence, thorough cleaning prior to any disinfection procedure is paramount.

There are different levels of disinfection:

- Low level disinfection (LLD): Elimination of most bacteria, some fungi and some viruses.
- Intermediate level disinfection: Elimination of most bacteria including mycobacteria, most fungi and some viruses but not bacterial spores.
- High level disinfection (HLD): Elimination of all viable pathogens apart from spores.

## Sterilisation

Elimination of all microbes including bacterial and fungal spores. This is usually achieved through autoclaving (using steam under high pressure) or exposing instruments to high temperatures; thus it is not suitable for US transducers. Current methods of sterilisation do not inactivate prions.

Chemical sterilisation by exposing medical devices to chemical agents such as peracetic acid, hypochloric acid, etc., is possible. Nevertheless, is it not considered to be fully equivalent to heat/steam sterilisation and chemicals may cause transducer surface damage. Furthermore, most of the agents used for chemical sterilisation are likely to pose a health hazard to both patients and staff through direct skin contact or inhalation.

## Recommendations

Worldwide there are an increasing number of infection prevention surveys and guidance documents available, most recently from the Australasian Society for Ultrasound in Medicine and the Australasian College for Infection Prevention and Control [20, 21]. Detailed Scottish guidance was published in 2016 [22–24], and subsequently adapted for Ireland [25]. Welsh guidance is available from 2014 but mainly focuses on endoscope decontamination [26]. References to hygiene can be found in 2016 Society and College of Radiographers and British Medical Ultrasound Society Guidelines for Professional Ultrasound Practice [27]. UK results of the large World Federation for Ultrasound in Medicine and Biology (WFUMB) survey were published in 2016 [28].

In Germany, Merz et al., like others, favours automated systems for high level disinfection, in particular devices using hydrogen peroxide (Trophon® EPR), now approved by the US Food and Drug Administration [29, 30]. Another important aspect of automated systems is the standardised and reproducible decontamination process thus avoiding operator-associated errors or variations. Ultraviolet (UV) light is less effective in eradicating microbes in comparison to hydrogen peroxide [31]. However, comparing different methods of decontamination is outside the scope of this publication. A publication by Rutala last year emphasises the need for HLD of all semi-critical and critical devices, already detailed in the original American guidance from 2008 [32, 33]. The American Institute of Ultrasound in Medicine (AIUM) also formulated guidance in 2014 [34] and French guidance and a survey were recently published [35, 36]. As previously mentioned, guidance from the European Federation of Societies for Ultrasound in Medicine and Biology (EFSUMB) in the interventional US setting is available [6], but no recent European Directive relating to this topic could be found.

### General principles

It is the responsibility of every US practitioner to ensure that cross-contamination risks are minimised. Any equipment used and the environment must be safe for all patients. General principles of infection prevention should be followed at all times.

#### Recommendation 1.1

- High standards of professional cleanliness such as good hand hygiene of the operator before and after every patient contact are essential.
- Thorough decontamination of US transducers and any equipment in direct patient contact before and after every patient, to the level required for specific procedures and in compliance with manufacturer specifications to avoid transducer surface damage, should be carried out. This includes regular decontamination of the US keyboard/console and any cables.
- Where possible, single use disposable equipment is preferable (biopsy needles, needle guides, etc.), eliminating the risk of inadequate cleaning and disinfection/sterilisation.
- Damaged US transducers should not be used as the risk of inadequate decontamination increases [10].
- Regular deep cleaning of the entire US equipment and the surrounding environment is essential.
- Use protective transducer covers dependent on the type of procedure. The use of transducer covers does not replace thorough decontamination, but merely reduces the contamination load.
- Adequate use of personal protective equipment as required by the procedure (non-invasive versus invasive) is mandatory.
- Use sterile US gel depending on procedure and patient’s risk of acquiring infections.
- Appropriate waste disposal is essential.

The management of patients with variant Creutzfeld-Jacob disease and other spongiforme encephalopathies is not covered by these best practice recommendations. US-practitioners must refer to specific national guidance.

### US transducer and other US equipment decontamination

#### Recommendation 2.1

The clinical environment and all deployed equipment must meet the infection prevention requirements of the respective procedure.

Should US be performed with handheld devices (tablets and mobile phones), these must be assessed and approved prior to preliminary use. The same cleaning and disinfection recommendations need to be followed as for normal units. Contamination of such devices should not be underestimated [37–39].

#### Recommendation 2.2

All US equipment in direct or indirect patient contact must be thoroughly cleaned and disinfected at the start of the examination and after every patient.

This includes the US transducer with handle, cable and transducer holder (as far as possible) as well as all additional devices which may be used during diagnostic or interventional procedures such as US fusion sensors/cables, needle guides (if reused), etc. Contamination of US equipment may be underestimated [40]. In particular, inadequately cleaned and disinfected needle guides have been associated with outbreaks of infection [41]. The use of single use needle guides is preferable to eliminate the risks associated with difficult to clean small bore devices.

The length of the drying time between cleaning and disinfection steps depends on the applied disinfectants/method used and no exact recommendations can be made. Regarding the choice of disinfectants, some disinfectants (in particular alcohol) may be ineffective in eliminating HPV type 16 [42], whilst also causing transducer surface damage [43], although alcohol still seems widely used in some countries [44].

### Non-critical US examinations: Transducer on intact surface skin

This applies only to procedures where no contact with body fluids exists and where no skin disease or known transmissible infections are present. In these circumstances the general consensus is that LLD is sufficient.

Decontamination steps necessary at the start of the examination and after every patient are as follows:

#### Recommendation 3.1

- Thorough cleaning of transducer: It is essential to remove all gel with soap and running water or detergent wipes prior to application of disinfectants. The use of detergents will aid removal of invisible gel remnants that disinfectants cannot penetrate and which may contain pathogens. Using dry paper to wipe transducers will remove some contamination [45, 46], however, this is not recommended as it is less effective than detergent wipes/soap and may scratch transducer surfaces.
- The transducer should be effectively dried: In order to avoid dilution of subsequently applied disinfection agents it is important to allow the transducer to dry. Application of disinfectants on a wet transducer will make them less effective or completely ineffective.
- Disinfection of US transducer: For this non-critical category, LLD can be achieved using wipes, foam or other approved agents with antibacterial, antiviral and antifungal properties. Products used should always be in compliance with manufacturers’ recommendations to avoid transducer surface damage.
- The transducer should be effectively dried: Following application of disinfectants, it is essential to allow sufficient time for the disinfectant to attain maximum effect.

### Critical and semi-critical US procedures

This refers to procedures where the transducer (with protective cover) is in contact with:

- Mucous membranes (all endo-cavity US)
- Any body fluids (all US guided interventional procedures including injections, tissue sampling, use in theatre)
- Infected/broken skin and wounds

The general consensus is that these procedures require HLD of US transducers including the handle [47]. An audit trail (detailed log) should be completed as evidence that thorough decontamination has been performed by accountable trained personnel. Care should be taken that storage of US transducers after HLD is adequate to avoid accidental contamination.

Decontamination steps necessary at the start of the examination and after every patient are as follows:

#### Recommendation 4.1

- The protective sheath should be carefully removed: It is important to avoid additional transducer contamination where possible.
- The transducer should be thoroughly cleaned: It is essential to remove all gel with soap and running water or detergent wipes prior to application of disinfectants. The use of detergents will aid removal of invisible gel remnants that disinfectants cannot penetrate and which may contain pathogens. Using dry paper to wipe transducers will remove some contamination [45, 46], however, this is not recommended as it is less effective than detergent wipes/soap and may scratch transducer surfaces.
- The transducer should be effectively dried: In order to avoid dilution of subsequently applied disinfection agents, it is important to allow the transducer to dry. Application of disinfectants on a wet transducer will make them less effective or completely ineffective.
- High level disinfection must be performed for all semi-critical and critical US procedures as persistent contamination following LLD has been demonstrated, even with transducer cover use [48–51]. Agents/methods used must be in compliance with manufacturers’ recommendations. One of the following may be chosen:
  - Approved manual multistep disinfectant wipes (validated for HLD)
  - Standardised automated validated systems (hydrogen peroxide, ultraviolet light)
  - Other approved procedures that have been validated for HLD including immersion bath
- The transducer should be effectively dried: It is essential to allow sufficient time for the disinfectant to attain maximum effect dependent on HLD method used.

### Transducer covers

Transducer covers are an integral part of infection prevention, as soiling of the transducer is substantially reduced leading to more effective post-procedure decontamination. However, the use of transducer covers does not eliminate the need for subsequent cleaning and disinfection as persistent contamination remains after cover removal [49–52]. Non-negligible contamination levels can also be detected after LLD when transducer covers are used, which is why HLD is essential [48].

The US practitioner is responsible for ensuring that only dedicated US transducer covers are used which are of adequate quality. Covers used should display the CE mark of quality testing or its equivalent. Barriers such as thin household cling film, plastic wraps or similar are not acceptable as product quality is not assured. Although some studies appear to show a lower perforation rate, the use of condoms as transducer covers is questionable [53, 54]. Even with dedicated transducer covers, perforation rates appear to be quite high, although there is a paucity of literature and newer materials may prove to be safer [55–57].

The following further recommendations are essential:

#### Recommendation 5.1

- Covers should always be strictly single-use.
- Appropriate covers need to be chosen for patients with latex allergies.
- The use of transducer covers is obligatory for all endo-cavity US, including trans-vaginal, trans-rectal, trans-oesophageal, and trans-bronchial US.
- Transducer covers must be used for all major and minor interventional procedures, whenever transducers may be in contact with body fluids such as blood, secretions, pus, etc. This includes all invasive interventions as well as injections, fine needle aspirations and transducer contact with infected or broken skin, eczema and wounds.
- Sterile transducer covers must be used for any invasive procedures detailed above. For all non-invasive examinations including endo-cavity ultrasound, sterile covers are recommended but not essential. Stocking only sterile covers may eliminate the risk of accidental use of non-sterile covers, but there are cost implications.

### Ultrasound gel

US gels are generally composed of a polymer to establish the desired viscosity, substances such as tri-ethanolamine to stabilise the pH, deionised water, a moisture retaining agent such as a glycol derivative, and often preservative agents. As bacteria are able to adjust their metabolism to a less favourable environment, these gel compounds are more than sufficient to allow bacterial survival and multiplication [58, 59].

Even sealed US gel bottles must not be assumed to be sterile unless clearly stated on packaging. Whilst the risk of infection transmission through contaminated US gel appears to be generally low, several outbreaks related to medical gels have been published [60–66]. Therefore, recommendations are as follows:

#### Recommendation 6.1

- Single use bottles are recommended rather than bottles that are refilled from larger containers. The latter poses a higher risk of contamination.
- Standard non-sterile bottles are sufficient if the transducer is in contact with intact skin only and in the absence of infections or other skin pathology, i.e., non-critical US examinations.
- Once opened, gel bottles should be used within a short period of time and ideally discarded at the end of the working day. Noting the opening date on bottles may be helpful.
- Care should be taken to avoid contact of the gel dispensing tip with the patient or other sources of contamination.
- Gels should be stored at room temperature. The multiplication of pathogens in gel bottles increases considerably when kept warm for patient comfort, thus turning bottle warmers into incubators [67]. Therefore, if gel warmers are used, only bottles for immediate use should be warmed.
- The regular decontamination of any bottle warmer facilities in use is essential whilst considering manufacturers guidance. Electrical devices should be unplugged and devices may need to cool down prior to decontamination.
- Gel bottles should not be stored upside down in warmers as the gel dispensing tip may become contaminated through patient contact or indeed through contact with pathogens surviving/multiplying at the bottom of warmers.
- Only dry bottle warmers should be used as any liquids will become even more easily contaminated [59, 68].

Particular consideration should be given to examinations and procedures where the choice of sterile gel is indicated:

#### Recommendation 6.2

- The use of sterile gel is highly recommended for all semi-critical and critical US procedures, such as transducer contact with mucous membranes, i.e., all endo-cavity US, contact with any body fluids, i.e., all major and minor US guided interventional procedures, and when scanning infected or broken skin and wounds.
- The use of sterile gel is strongly advised outside as well as inside transducer covers due to high reported transducer cover perforation rates and possible porosity [55–57]. A new sachet should be opened for every patient but the same sachet can be used for gel inside and outside the probe cover.

## Conclusion

Published evidence highlights contamination risks of US transducers, even with the use of transducer covers, and after LLD. Although published cases of proven infection transmission through US are limited at present, this should not induce complacency as the true rates are unknown. Many practitioners do not seem to adhere to or be aware of basic infection prevention procedures as shown in several recent surveys. This emphasises the need for new and improved standards.

With publication of these best practice recommendations the ESR US WG aims to stress:

- The importance of basic personal and environmental hygiene.
- The need for thorough equipment decontamination as appropriate for the respective examination or intervention.
- Risk reduction through appropriate use of transducer covers and sterile gel where indicated.

US practitioners cannot always know which patients carry transmissible pathogens or who may be susceptible to acquiring infections; hence, high standards of infection prevention and control will ensure that all patients are kept safe.

These recommendations should be reviewed and locally adapted in accordance with respective national guidance, where available, to suit the respective clinical environment. Three flow charts (see Appendix) are included with this set of recommendations to assist US practitioners and others in achieving best practice.

Published evidence is limited and partly dated, therefore publication of evidence-based guidance at present is challenging, but needed. We hope that more emerging evidence will make this possible in the near future through the conduct of audits, surveys and even clinical trials where possible.

The ESR US WG is already in discussions with the European Coordination Committee of the Radiological Electromedical and Healthcare IT Industry (COCIR) and the ESR Patient Advisory Group. A joint session with discussion was organised at the European Congress of Radiology in March 2017 and both groups were consulted in the process of writing these recommendations. It would be desirable for manufacturers of US equipment, transducer covers, US gel and cleaning/disinfection consumables/equipment to collaborate further with US practitioners and patient representatives to improve standards. Additional research studies on the susceptibility of pathogens to practicable, low/non-toxic and affordable disinfection measures are needed, and how these can be best applied in the context of US.

We recognise that implementing thorough US decontamination protocols will necessitate an initial investment and increasing ongoing consumable costs as well as additional staff training. However, we believe that the implementation of clear recommendations will reassure patients, and contribute to the quality of their care.

## Appendix

**Ultrasound equipment decontamination**

• **High standards of professional hygiene must be ensured at all times**, including good operator hand hygiene before/after patient contact, adequate use of personal protective equipment, probe covers, etc., as required. Appropriate clinical waste disposal protocols must be in place.

• The clinical environment and all deployed equipment must meet the infection prevention requirements of the respective procedure. Should US be performed on **handheld devices** (tablets and mobile phones) these must be assessed and approved prior to preliminary use and disinfected before and after each episode of deployment.

• **All US equipment in direct or indirect patient contact must be thoroughly cleaned and disinfected before the first patient and after every patient. This includes the US transducer, cable and transducer holder** (as far as possible) as well as **all additional devices**, which may be used during diagnostic or interventional procedures, such as US **fusion sensors/cables, needle guides**, etc. Regular deep cleaning of the entire US machine and environment is essential.

**Protective ultrasound transducer covers**

• The **US practitioner is responsible for ensuring that only dedicated US transducer covers of adequate quality are used**. Practitioners should check that all covers chosen display the CE mark of quality testing or its equivalent.

• Barriers such as thin household cling film/plastic wraps, etc., are **not acceptable** as these are easily perforated and the product quality is not assured

**Ultrasound gel**

• Practitioners cannot assume that US gel is free of pathogens unless it is clearly labelled as “sterile”.

• **Pathogens can survive and multiply within gel**. Outbreaks due to this have been documented.

• If used for patient comfort, **bottle warmers should be regularly cleaned and disinfected**. It is not advisable to keep bottles warmed for long periods as this will aid multiplication of microbes. Bottles should not be kept in warmers upside down as the dispensing tip may become contaminated by accidental patient contact or from a previously inserted bottle. Warming in an immersion bath is not recommended as fluids easily become contaminated.

## Footnotes

1 The Spaulding Classification was proposed by Earle Spaulding in 1939 and is still widely used in the literature and guidance documents

## References

[1] Sartoretti T, Sartoretti E, Bucher C, Doert A, Binkert C, Hergan K, Meissnitzer M, Froehlich J, Kolokythas O, Matoori S, Orasch C, Kos S, Sartoretti-Schefer S, Gutzeit A (2017) Bacterial contamination of ultrasound probes in different radiological institutions before and after specific hygiene training: do we have a general hygienical problem? Eur Radiol. 10.1007/s00330-017-4812-1 10.1007/s00330-017-4812-1 28374081

[2] Kramer A, Schwebke I, Kampf G (2006). How long do nosocomial pathogens persist on inanimate surfaces? A systematic review. BMC Infect Dis.

[3] Paintsil E, Binka M, Patel A, Lindenbach BD, Heimer R (2014). Hepatitis C virus maintains infectivity for weeks after drying on inanimate surfaces at room temperature: implications for risks of transmission. J Infect Dis.

[4] Lesourd F, Izopet J, Mervan C, Payen JL, Sandres K (2000). Transmissions of hepatitis C virus during the ancillary procedures for assisted conception. Hum Reprod.

[5] Ferhi K, Rouprêt TM, Mozer P, Ploussard G, Haertig A, De La Taille A (2013) Hepatitis C transmission after prostate biopsy. Case Rep e797248 https://wwwncbinlmnihgov/pmc/articles/PMC3600139/pdf/CRIMUROLOGY2013-797248pdf Accessed 3 July 2017 10.1155/2013/797248 PMC3600139 23533934

[6] Lorentzen T, Nolsøe CP (2015). EFSUMB guidelines on interventional ultrasound (INVUS), part I. Ultraschall Med.

[7] Nyhsen CM, Humphreys H, Nicolau C, Mostbeck G, Claudon M (2016) Infection prevention and ultrasound probe decontamination practices in Europe: a survey of the European society of radiology. Insights Imaging 2016 Dec;7(6):841-847 10.1007/s13244-016-0528-z PMC5110482 27778309

[8] Koibuchi H, Kotani K, Taniguchi N (2013). Ultrasound probes as a possible vector of bacterial transmission. Med Ultrason.

[9] Bancroft EA, English L, Terashita D, Yasuda L (2013). Outbreak of Escherichia Coli infections associated with a contaminated transesophageal echocardiography probe. Infect Control Hosp Epidemiol.

[10] Seki M, Machida H, Yamagishi Y, Yoshida H, Tomono K (2013). Nosocomial outbreak of multidrug-resistant Pseudomonas Aeruginosa caused by damaged transesophageal echocardiogram probe used in cardiovascular surgical operations. J Infect Chemother.

[11] Paz A, Bauer H, Potasman I (2001). Multiresistant Pseudomonas Aeruginosa associated with contaminated transrectal ultrasound. J Hosp Infect.

[12] Leroy S (2013). Infectious risk of endovaginal and transrectal ultrasonography: systematic review and meta-analysis. J Hosp Infect.

[13] Leroy S, M’Zali F, Kann M, Weber D, Smith D (2014). Impact of vaginal-rectal ultrasound examinations with covered and low-level disinfected transducers on infectious transmissions in France. Infect Control Hosp Epidemiol.

[14] Bénet T, Ritter J, Vanhems P (2014). Risk of human immunodeficiency virus and hepatitis C virus infection related to endocavitary ultrasound probe exposure in France. Infect Control Hosp Epidemiol.

[15] Spaulding EH, Lawrence C, Block SS (1968). Chemical disinfection of medical and surgical materials. Disinfection, sterilization, and preservation.

[16] Lemos MA, Silva JBG, Braga ACS (2014). Acupuncture needles can carry Hepatitis C Virus. Infect Control Hosp Epidemiol.

[17] Encyclopædia Britannica (2017) https://www.britannica.com/biography/Typhoid-Mary Accessed 19 June 2017

[18] Lodeta B, Trkulja V (2014). Septic complications and hospital admissions after transrectal ultrasound-guided prostate biopsy: incidence rates and outcomes. Int Urol Nephrol.

[19] Campeggi A, Ouzaid I, Xylinas E, Lesprit P, Hoznek A, Vordos D, Abbou CC, Salomon L, de la Taille A (2014). Acute bacterial prostatitis after transrectal ultrasound-guided prostate biopsy: epidemiological, bacteria and treatment patterns from a 4-year prospective study. Int J Urol.

[20] Westerway SC, Basseal JM (2017) Advancing infection control in Australasian medical ultrasound practice. AJUM 20(1) http://onlinelibrarywileycom/doi/101002/ajum12046/epdf Accessed 3 July 2017 10.1002/ajum.12046 PMC8409851 34760467

[21] Guidelines for Reprocessing Ultrasound Transducers by the Australasian Society for Ultrasound in Medicine and the Australasian College for Infection Prevention and Control (2017) AJUM 20 (1) http://onlinelibrary.wiley.com/doi/10.1002/ajum.12042/epdf Accessed 3 July 2017 10.1002/ajum.12042 PMC8409821 34760468

[22] Health Facilities Scotland Decontamination Services (2016) NHS Scotland guidance for decontamination of semi-critical ultrasound probes; semi-invasive and non-invasive ultrasound probes http://wwwhpsscotnhsuk/documents/hai/infectioncontrol/guidelines/NHSScotland-Guidance-for-Decontaminationof-Semi-Critical-Ultrasound-Probespdf Accessed 2 July 2017

[23] Health Protection Scotland NHS National Services Scotland (2016) Decontamination procedure for high level disinfection of semi-critical ultrasound probes (semi-invasive and non-invasive): using a manual (chlorine dioxide) multiwipe system. RES-183-4 HPS/HFS Version 10 http://wwwhpsscotnhsuk/resourcedocumentaspx?id=5732 Accessed 12 July 2017

[24] Health Protection Scotland NHS National Services Scotland (2016) Decontamination Procedure for high level disinfection (HLD) of Semi-critical Ultrasound Probes (Semi-invasive and Non-invasive): using ultraviolet C light http://www.hps.scot.nhs.uk/resourcedocument.aspx?id=5731 Accessed 12 July 2017

[25] Irish Health Service Executive (HSE) Quality Improvement Division - Decontamination Safety Programme (2017) HSE guidance for decontamination of semi‐critical ultrasound probes; Semi‐invasive and Non‐invasive Ultrasound Probes QPSD-GL-028-1 http://wwwhseie/eng/about/Who/QID/nationalsafetyprogrammes/decontamination/Ultrasound-Probe-Decontamination-Guidance-Feb-17pdf Accessed 12 July 2017

[26] NHS Wales Shared Services Partnership – Specialist Estates Services (2014) Welsh Health Technical Memorandum 01–06 Decontamination of flexible endoscopes Part C: Operational management (Including guidance on non-channelled endoscopes and ultrasound probes) http://www.wales.nhs.uk/sites3/Documents/254/WHTM%2001%2D06%20Part%20C.pdf Accessed 12 July 2017

[27] SCoR and BMUS Guidelines for Professional Ultrasound Practice (2016) https://www.sor.org/sites/default/files/document-versions/bmus_scor_ultrasound_guidelines.pdf Accessed 2 July 2017

[28] Westerway SC, Basseal JM2 (2017) The ultrasound unit and infection control - are we on the right track? Ultrasound 25(1):53-57. 10.1177/1742271X16688720 PMC5308390 28228825

[29] Merz E (2016). Is transducer hygiene sufficient when vaginal probes are used in the clinical routine?. Ultraschall Med.

[30] Ryndock E, Robison R, Meyers C (2015). Susceptibility of HPV16 and 18 to high level disinfectants indicated for semi-critical ultrasound probes. J Med Virol.

[31] Havill NL, Moore BA, Boyce JM (2012). Comparison of the microbiological efficacy of hydrogen peroxide vapor and ultraviolet light processes for room decontamination. Infect Control Hosp Epidemiol.

[32] Rutala WA, Weber D (2016). Reprocessing semicritical items. Am J Infect Control.

[33] Rutala WA, Weber DJ, and the Healthcare Infection Control Practices Advisory Committee (HICPAC) (2008) Guideline for disinfection and sterilization in healthcare facilities. Available: http://www.cdc.gov/hicpac/pdf/guidelines/disinfection_nov_2008.pdf Accessed Jan 29 2016

[34] American Institute of Ultrasound in Medicine (aium, 2014) Guidelines for cleaning and preparing external- and internal-use ultrasound probes between patients. http://wwwaiumorg/officialstatements/57 Accessed 29 Jan 2016

[35] Haut Conseil de la santé publique: Avis relatif à la désinfection des sondes à échographie endocavitaire (2016) http://www.hcsp.fr/explore.cgi/avisrapportsdomaine?clefr=561 Accessed 12 July 2017

[36] Réseau national de prevention des infections associées aux soins – Groupe d’évaluation des pratiques en hygiène hospitalière (2016) Enquête relative aux pratiques d'hygiène appliquées aux sondes à échographie endovaginale http://www.grephh.fr/PDF/Sondes/Resultats_enquete_sondeEE_octobre-2016.pdf Accessed 12 July 2017

[37] Ulger F, Dilek A, Esen S, Sunbul M, Leblebicioglu H (2015). Are healthcare workers' mobile phones a potential source of nosocomial infections? Review of the literature. J Infect Dev Ctries 29.

[38] Cavari Y, Kaplan O, Zander A, Hazan G, Shemer-Avni Y, Borer A (2016). Healthcare workers mobile phone usage: a potential risk for viral contamination. Surveillance pilot study. Infect Dis (Lond).

[39] Pillet S, Berthelot P, Gagneux-Brunon A, Mory O, Gay C, Viallon A, Lucht F, Pozzetto B, Botelho-Nevers E (2016). Contamination of healthcare workers' mobile phones by epidemic viruses. Clin Microbiol Infect.

[40] Keys M, Sim B, Thom O, Tunbridge M, Barnett A, Fraser J (2015). Efforts to attenuate the spread of infection (EASI): a prospective, observational multicenter survey of ultrasound equipment in Australian emergency departments and intensive care units. Crit Care Resusc.

[41] Gillespie JL, Arnold KE, Noble-Wang J, Jensen B, Arduino M, Hageman J (2007). Outbreak of Pseudomonas Aeruginosa infections after transrectal ultrasound-guided prostate biopsy. Urology.

[42] Meyers J, Ryndock E, Conway MJ, Meyers C, Robison R (2014). Susceptibility of high-risk human papillomavirus type 16 to clinical disinfectants. J Antimicrob Chemother.

[43] Koibuchi H, Fujii Y, Kotani K (2011). Degradation of ultrasound probes caused by disinfection with alcohol. J Med Ultrason.

[44] Häggström M, Spira J, Edelstam G (2015). Transducer hygiene: comparison of procedures for decontamination of ultrasound transducers and their use in clinical practice. J Clin Ultrasound.

[45] Ejtehadi F, Ejtehadi F, Teb JC, Arasteh MM (2014). A safe and practical decontamination method to reduce the risk of bacterial colonization of ultrasound transducers. J Clin Ultrasound.

[46] Mullaney PJ, Munthali P (2007). How clean is your probe? Microbiological assessment of ultrasound transducers in routine clinical use, and cost-effective ways to reduce contamination. Clin Radiol.

[47] Ngu A, McNally G, Patel D, Gorgis V, Leroy S, Burdach J (2015). Reducing transmission risk through high-level disinfection of transvaginal ultrasound transducer handles. Infect Control Hosp Epidemiol.

[48] M’Zali F, Bounizra C, Leroy S, Mekki Y, Quentin-Noury C, Kann M (2014). Persistence of microbial contamination on transvaginal ultrasound probes despite low-level disinfection procedure. PLoS One.

[49] Ma ST, Yeung AC, Chan PK, Graham CA (2013). Transvaginal ultrasound probe contamination by the human papillomavirus in the emergency department. Emerg Med J.

[50] Casalegno JS, Le Bail CK, Eibach D, ValdeyronML LG, Jacquemoud H (2012). High risk HPV contamination of endocavity vaginal ultrasound probes: an underestimated route of nosocomial infection?. PLoS One.

[51] Kac G, Podglajen I, Si-Mohamed A, Rodi A, Grataloup C, Meyer G (2010) Evaluation of ultraviolet C for disinfection of endocavitary ultrasound transducers persistently contaminated despite probe covers 31(2):165-70 10.1086/649794 20025531

[52] Storment JM, Monga M, Blanco JD (1997). Ineffectiveness of latex condoms in preventing contamination of the transvaginal ultrasound transducer head. South Med J.

[53] Amis S, Ruddy M, Kibbler CC, Economides DL, MacLean AB (2000). Assessment of condoms as probe covers for transvaginal sonography. J Clin Ultrasond.

[54] Rooks VJ, Yancey MK, Elg SA, Brueske L (1996). Comparison of probe sheaths for endovaginal sonography. Obstet Gynecol.

[55] Milki AA, Fisch JD (1998). Vaginal ultrasound probe cover leakage: implications for patient care. Fertil Steril.

[56] Masood J, Voulgaris S, Awogu O, Younis C, Ball AJ, Carr TW (2007). Condom perforation during transrectal ultrasound guided (TRUS) prostate biopsies: a potential infection risk. Int Urol Nephrol.

[57] Hignett M, Claman P (1995). High rates of perforation are found in endovaginal ultrasound probe covers before and after oocyte retrieval for in vitro fertilization-embryo transfer. J Assist Reprod Genet.

[58] Muradali D, GoldWL PA, Wilson S (1995). Can ultrasound probes and coupling gel be a source of nosocomial infection in patients undergoing sonography? An in vivo and in vitro study. Am J Roentgenol.

[59] Oleszkowicz SC, Chittick P (2012). Infections associated with use of ultrasound transmission gel. Proposed guidelines to minimize risk. Infect Control Hosp Epidemiol.

[60] Cheng A, Sheng W-H, Huang Y-C, Sun H-Y, Tsai Y-T, Chen M-L (2016). Prolonged postprocedural outbreak of Mycobacterium Massiliense infections associated with ultrasound transmission gel. Clin Microbiol Infect.

[61] Chittick P, Russo V, SimsMet al (2012) Outbreak of Pseudomonas Aeruginosa respiratory tract infections in cardiovascular surgery associated with contaminated ultrasound gel used for transesophageal echocardiography—Michigan, December 2011–January 2012. MMWR Morb Mortal Wkly Rep 61:262–264 22513528

[62] Olshtain-Pops K, Block C, Temper V (2011). An outbreak of Achromobacter xylosoxidans associated with ultrasound gel used during transrectal ultrasound guided prostate biopsy. J Urol.

[63] Jacobson M, Wray R, Kovach D, Henry D, Speert D, Matlow A (2006). Sustained endemicity of Burkholderia cepacia complex in a pediatric institution, associated with contaminated ultrasound gel. Infect Control Hosp Epidemiol.

[64] Hutchinson J, Runge W, Mulvey M (2004). Burkholderia cepacia infections associated with intrinsically contaminated ultrasound gel: the role of microbial degradation of parabens. Infect Control Hosp Epidemiol.

[65] Weist K, Wendt C, Petersen L, Versmold H, Ruden H (2000). An outbreak of pyodermas among neonates caused by ultrasound gel contaminated with methicillin-susceptible Staphylococcus Aureus. Infect Control Hosp Epidemiol.

[66] Gaillot O, Maruéjouls C, Abachin E (1998). Nosocomial outbreak of Klebsiella Pneumoniae producing SHV-5 extended-spectrum beta-lactamase, originating from a contaminated ultrasonography coupling gel. J Clin Microbiol.

[67] Westerway S, Basseal JM, Brockway A, Hyett JA, Carter DA (2016). Potential risks associated with an ultrasound examination – a bacterial perspective. J Ultrasound Med Biol.

[68] Muyldermans G, de Smet F, Pierard D (1998). Neonatal infections with Pseudomonas Aeruginosa associated with a water-bath used to thaw fresh frozen plasma. J Hosp Infect.

